# No Effects of Non-invasive Brain Stimulation on Multiple Sessions of Object-Location-Memory Training in Healthy Older Adults

**DOI:** 10.3389/fnins.2017.00746

**Published:** 2018-01-10

**Authors:** Nadine Külzow, Angelica Vieira Cavalcanti de Sousa, Magda Cesarz, Julie-Marie Hanke, Alida Günsberg, Solvejg Harder, Swantje Koblitz, Ulrike Grittner, Agnes Flöel

**Affiliations:** ^1^Charité - Universitätsmedizin Berlin, Freie Universität Berlin, Humboldt-Universität zu Berlin and Berlin Institute of Health, Neurocure Cluster of Excellence, Berlin, Germany; ^2^Clinical Research Unit, Berlin Institute of Health, Berlin, Germany; ^3^Charité - Universitätsmedizin Berlin, Department of Biostatistics and Clinical Epidemiology, Freie Universität Berlin, Humboldt-Universität zu Berlin and Berlin Institute of Health, Berlin, Germany; ^4^Charité - Universitätsmedizin Berlin, Freie Universität Berlin, Humboldt-Universität zu Berlin and Berlin Institute of Health, Center for Stroke Research, Berlin, Germany; ^5^Department of Neurology, University Medicine Greifswald, Greifswald, Germany

**Keywords:** aging, visuospatial memory, episodic memory, associative learning, transcranial direct current stimulation, cognitive training, transfer effects, consolidation

## Abstract

Object-location memory (OLM) is known to decline with normal aging, a process accelerated in pathological conditions like mild cognitive impairment (MCI). In order to maintain cognitive health and to delay the transition from healthy to pathological conditions, novel strategies are being explored. Tentative evidence suggests that combining cognitive training and anodal transcranial direct current stimulation (atDCS), both reported to induce small and often inconsistent behavioral improvements, could generate larger or more consistent improvements or both, compared to each intervention alone. Here, we explored the combined efficacy of these techniques on OLM. In a subject-blind sham-controlled cross-over design 32 healthy older adults underwent a 3-day visuospatial training paired with either anodal (20 min) or sham (30 s) atDCS (1 mA, temporoparietal). Subjects were asked to learn the correct object-location pairings on a street map, shown over five learning blocks on each training day. Acquisition performance was assessed by accuracy on a given learning block in terms of percentage of correct responses. Training success (performance on last training day) and delayed memory after 1-month were analyzed by mixed model analysis and were controlled for gender, age, education, sequence of stimulation and baseline performance. Exploratory analysis of atDCS effects on within-session (online) and between-session (offline) memory performance were conducted. Moreover, transfer effects on similar trained (visuospatial) and less similar (visuo-constructive, verbal) untrained memory tasks were explored, both immediately after training, and on follow-up. We found that atDCS paired with OLM-training did not enhance success in training or performance in 1-month delayed memory or transfer tasks. In sum, this study did not support the notion that the combined atDCS-training approach improves immediate or delayed OLM in older adults. However, specifics of the experimental design, and a non-optimal timing of atDCS between sessions might have masked beneficial effects and should be more systematically addressed in future studies.

## Introduction

Remembering the place of an object (object-location memory, OLM) is crucial for adapting to changing environments in every-day life. However, this ability is known to decline during aging (Hedden and Gabrieli, 2004; Kessels et al., 2007) and may represent an incipient marker of neurodegenerative disease (Iachini et al., 2009). Advanced age increases risk of cognitive impairment and other age-related diseases (Salthouse, 2010; Niccoli and Partridge, 2012; Harada et al., 2013). Hence, early preventative strategies aiming to activate cognitive and brain resources in order to retain cognitive health, autonomy, and a better quality of life (Depp and Jeste, 2006) are of paramount importance. A variety of approaches have been investigated for its therapeutic and neuro-enhancing potential, including cognitive training, dietary regimes, physical training, use of pharmacological agents, as well as non-invasive brain stimulation (Perceval et al., 2016).

Cognitive training (CT) usually involves strengthening of neural networks through repeated co-activation of specific neurocognitive circuits active during task performance (Santarnecchi et al., 2015). The literature indicates that CT is beneficial for older adults' memory, but gains are much smaller than in young subjects (c.f. Passow et al., 2017). Moreover, evidence for generalizing effects that go beyond trained domain (transfer effects) is scarce and inconsistent (Jaeggi et al., 2014). In addition, CT is generally time-consuming (applied over weeks) and might therefore suffer from low motivation and treatment adherence over time (Martin et al., 2013; Elmasry et al., 2015). Importantly, CT may be combined with and boosted by any of the other interventions, specifically non-invasive brain stimulation, offering a convenient application to further promote training effects (Prehn and Floel, 2015; Au et al., 2017).

Anodal transcranial direct current stimulation (atDCS) is a non-invasive and painless technique increasingly used to modulate memory in aging (Bennabi et al., 2014). The rationale behind its use is the potential of atDCS to increase cortical excitability in targeted brain areas by subthreshold alteration of resting membrane potential (Nitsche et al., 2003; Giordano et al., 2017) and a modulation of glutamatergic neurotransmission which promote mechanisms of long-term-potentiation (LTP; Stagg and Nitsche, 2011). Of note, in a previous study, we demonstrated no impact of atDCS on immediate recall in OLM, but observed an improvement with 1-week delay after cessation of a single stimulation period (Floel et al., 2012). Consequently, and in accordance with others a consolidation mechanism susceptible to atDCS was suggested (Reis et al., 2009, 2015; Prehn and Floel, 2015; Perceval et al., 2016; Sandrini et al., 2016). Hence, larger or more sustained improvements may be induced by repeated applications of a combined atDCS-training approach (e.g., Hsu et al., 2015; Nilsson et al., 2015). This principle has been nicely laid out in Holland et al. (2011) suggesting that small gains would accumulate by each bout of stimulation on consecutive days.

Previous studies in the motor domain support this idea (Reis et al., 2009, 2015). With regard to memory training, most recent evidence stems from studies combing working memory training and atDCS (Passow et al., 2017). However, the findings have not been unequivocal. While some studies demonstrated a small but significant positive effect of atDCS over the course of training relative to sham (Park et al., 2014; Jones et al., 2015; Au et al., 2016), others did not (Lally et al., 2013; Stephens and Berryhill, 2016; Nilsson et al., 2017). Moreover, little is known about a putative synergistic effect of combined intervention in episodic memory in older adults (Prehn and Floel, 2015; Bartrés-Faz and Vidal-Piñeiro, 2016; Perceval et al., 2016; Birba et al., 2017; Passow et al., 2017). Such synergistic effect would predict greater, prolonged or more persistent improvements when interventions are applied together compared to each intervention alone (see also Ditye et al., 2012). Most notably there is only one published training-plus-atDCS study of Cotelli et al. (2014) on age-sensitive hippocampus-dependent associative memory like OLM. In this study, patients with Alzheimer's disease underwent a 10-session face-naming association memory training paired with left dorsolateral prefrontal cortex (LDPFC) stimulation. Memory did not benefit from atDCS relative to sham, possibly due to substantial inter-individual variability in degree of cognitive decline and brain organization, which may thus have altered brain responsiveness to atDCS.

What may account for the inconsistent findings across studies so far? A variety of factors have been discussed, such as age, gender, education, health status, genetic background, brain state, baseline performance, but also mood, motivation, activation, or quality of sleep (e.g., Krause and Cohen Kadosh, 2014; Santarnecchi et al., 2015; Hsu et al., 2016). Given the high functional relevance of OLM, which is vulnerable to decline during aging (Postma et al., 2008; Shih et al., 2012), and our promising finding after a single session application of atDCS delivered during a visuospatial task (Floel et al., 2012) we set out to assess the impact of a combined atDCS-OLM-training protocol. Therefore, healthy older subjects underwent an OLM-training on 3 consecutive days in a sham-controlled cross-over design. Performance immediately after the protocol (training success, primary outcome) and memory performance after 1 month (delayed memory, secondary outcome) were tested and controlled for main potential modulating factors (covariates: gender, age, education, sequence of stimulation and baseline performance). Moreover, interventions which are able to induce more generalized cognitive effects are of utmost behavioral relevance. Thus, we explored the impact of atDCS vs. sham stimulation on performance on trained (similar OLM task) and untrained (visuo-constructive and verbal) memory functions (transfer).

## Materials and methods

### Subjects

Healthy older adults between 50 and 90 years were recruited via advertisements in Berlin, Germany. Subjects were pre-screened by a structured phone interview for major exclusion criteria such as history of epilepsy or metal implants. Individuals that passed pre-screening underwent an on-site medical and neuropsychological screening, and a structural magnetic resonance imaging (MRI) to ascertain the following inclusion criteria: (1) native German language speaker; (2) no current intake of medication that affect the central nervous system (e.g., antipsychotics or antidepressants); (3) normal routine medical and neurological examinations; (4) no recreational drug use; and (5) no cognitive impairment as assessed by CERAD screening test (Consortium to Establish a Registry for Alzheimer's Disease test battery; Memory Clinic Basel, www.memoryclinic.ch). Specifically, results of CERAD memory scales had to be within 1 SD of age/education norms and Mini Mental State Examination ≥26 points (Folstein et al., 1975). Then, subjects completed a comprehensive baseline assessment. Baseline tests comprised cognitive status obtained from standard neuropsychological tests, and non-cognitive functions acquired from standardized questionnaires (for details see Table 1). From 56 subjects that were screened, 19 declined participation because of time constraints and one did not met inclusion criteria. From the remaining 36 subjects, four subjects had to be excluded due to abnormal MRI findings (*n* = 2) and technical problems during training sessions (*n* = 2), thus leaving 32 healthy older subjects (mean age (SD) in years, [range]: 68 (7), [53–79], 22 females) for analysis. All subjects gave written informed consent before study-specific procedures and received a reimbursement for participation. The study was approved by the Ethics Committee of the Charité - Universitätsmedizin Berlin, Germany, was conducted in accordance with the declaration of Helsinki, and was registered at https://clinicaltrials.gov/ (NCT02110056).

**Table 1:** Demographic, non-cognitive, and cognitive baseline characteristics for healthy older adults grouped according to stimulation (atDCS, sham) applied in the 1st study block.

	**atDCS Mean (SD)**	**Sham Mean (SD)**
N (women)	16 (11)	16 (11)
Age (years)	69 (6)	67 (8)
Education (no. of years)^a^	15 (3)	16 (3)
ApoE genotype ε4 allele carriers (N; %)	6^b^; 20%	12; 37%
**NON-COGNITIVE CHARACTERISTICS**		
Depression: BDI^a^	2.9 (2.4)	3.7 (3.1)
Quality of life: WHOQoL (overall score)^a^	76.6 (11.1)	73.3 (17.6)
Sleep: PSQI^b^	5.3 (2.6)	5.1 (3.6)
Coping with Stress: SVF: • positive strategies	13 (3.5)	19.1 (21.5)
• negative strategies	8.1 (3.3)	13.1 (23.2)
Motivation: NFC^c^	36 (22.0)	49.9 (34.2)
**COGNITIVE DOMAINS**		
MMSE	29.1 (1.3)	29.1 (1.3)
CERAD • word recall	7.9 (1.9)	8.3 (1.8)
• figures recall	9.6 (2.6)	9.3 (1.8)
Digit span • forward^a^	8.9 (1.7)	8.6 (2.1)
• backwards^a^	6.9 (2.4)	6.3 (1.9)
TMT-A (sec)	42 (14)	43 (14)
TMT-B (sec)	81 (26)	75 (28)
Fluency: • s-words	17.7 (4.7)	17.4 (5)
• category animals	23.9 (6.7)	23.2 (5.2)
• sport-fruits	15.3 (2.2)	15 (3.1)
TAP: • Inhibition (Go/NoGo; median in ms)	414.5 (118.7)	464.8 (78.9)
• Alertness	0.01 (0.13)	0.06 (0.08)
MWT	32.2 (2.0)	33.7 (1.2)

Data are given as mean (SD). In some parameters N is reduced due to missing data:

a *N = 31*,

b *N = 30*,

c *N = 29*.

*ApoE ε4 allele: Apolipoprotein E-DNA was extracted from whole blood using a blood mini-kit (Qiagen, Hilden, Germany) and genotyping on coded samples was performed by the lab of Prof. Dr. Dan Rujecscu, University Halle, Germany, procedure is described in more detail in Kerti et al. (2013). BDI, Becks depression inventory (Hautzinger et al., 2001); WHOQoL, WHO Quality of life (Angermeyer et al., 2000); SVF120, stress coping strategies—habitual form (Erdmann and Janke, 2008); PSQI, habitual sleep score (Pittsburgh Sleep Quality, Buysse et al., 1989); NFC, Need for Cognition (Bless et al., 1994); MMSE, Mini Mental State Examination scores (Folstein et al., 1975); CERAD, Consortium to Establish a Registry for Alzheimer's Disease test battery (Memory Clinic Basel, www.memoryclinic.ch); Digit span (Härting et al., 2000); TMT, Trail Making Test (Tombaugh, 2004); Fluency, Regensburger Verbal Fluency Test (Aschenbrenner et al., 2000); TAP, computerized test battery to test attention (Zimmermann et al., 2002); MWT, Multiple-Choice Vocabulary Intelligence Test (Lehrl, 2005)*.

### Experimental design

In this subject-blind, placebo-controlled, cross-over study all subjects were tested in two blocks. Each block comprised a 3-day visuospatial OLM-training, paired with either anodal or sham tDCS (combined intervention of atDCS+training and sham+training is abbreviated in the following to “atDCS” and “sham”). Order of stimulation (atDCS vs. sham) were pseudo-randomized and controlled for age and gender, with 16 subjects receiving atDCS first and 16 subjects receiving sham first. Additionally, sequence of intervention was balanced and separated by 3 months to prevent carry-over effects. Before (pre-training) and 1-day and 1-month after training (follow-up measurements FU1 and FU2), three other tasks to test trained (one task) and untrained (two tasks) memory functions were employed (transfer tasks). On average, subjects that started with atDCS first were comparable to subjects with sham first in terms of age, gender, distribution of ApoE ε4 allele carriers (a polymorphism that have been previously implicated in memory outcome, e.g., Wisdom et al., 2011; Matura et al., 2014), or baseline cognitive performance (see Table 1 for details). An exception was found for estimator of premorbid intelligence (German version of Multiple-Choice Vocabulary Intelligence Test, MWT, Lehrl, 2005). Here, subjects with sham first scored on average 1.5 points [correct words, mean (SD): 33.7 (1.2) vs. 32.2 (2.0)] higher than subjects in the atDCS group prior to intervention.

### Training task

**Figure 1:**
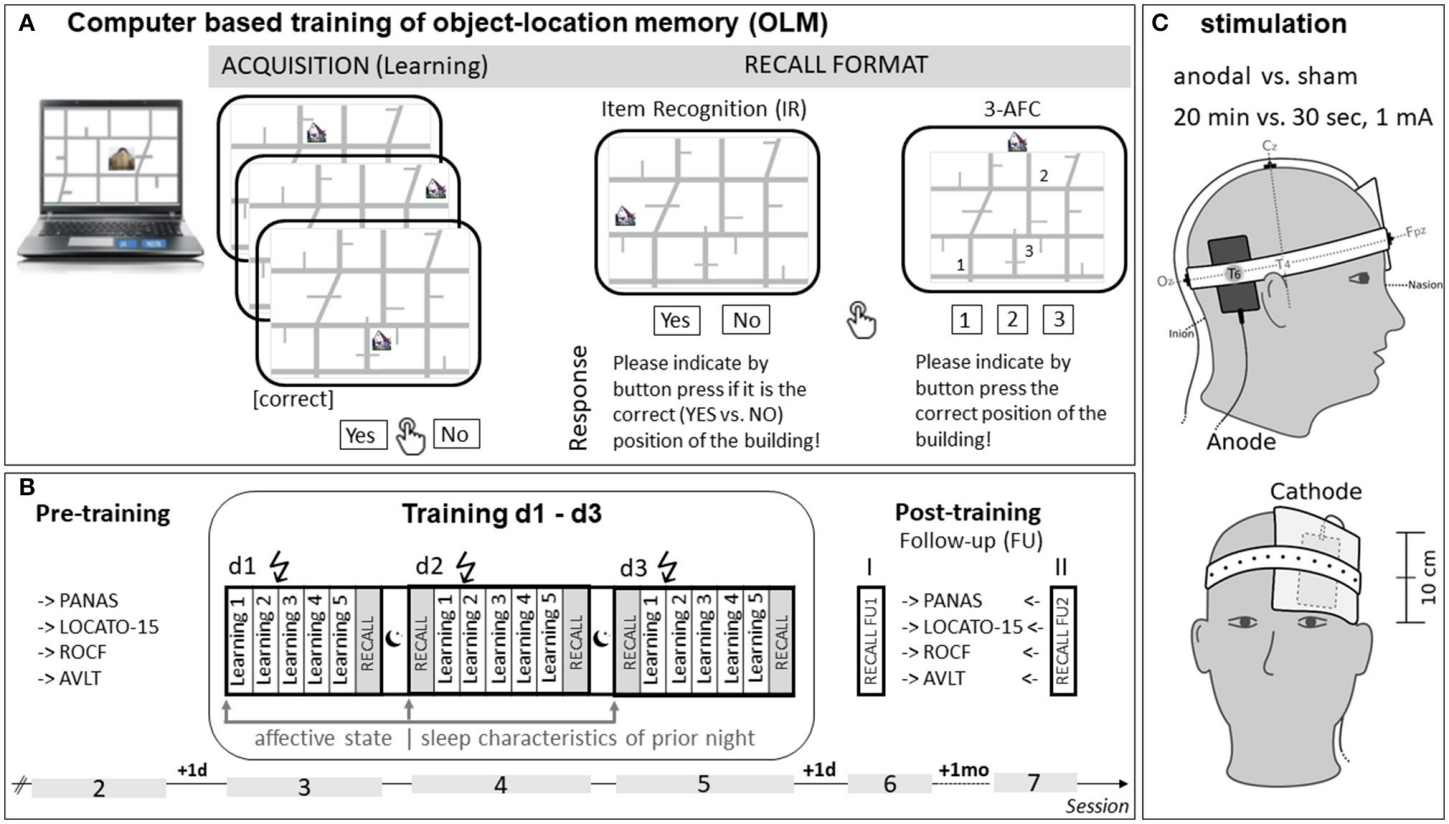
Study overview. **(A)** Schematic of the associative object-location learning paradigm (LOCATO). During acquisition, each trial comprised a picture of a schematized street map with one building. Buildings (objects) occurred on one correct and 10 incorrect positions (locations). Subjects had to learn correct object-location-pairings over the course of multiple learning blocks and indicate (button press) in each trial, whether a building was in a “correct” location (Yes or NO). Memory for object-location-associations were assessed by two different cued recall tests [item recognition (IR), 3-alternative-forced choice test (3-AFC)]. During IR correct object-location-associations were intermixed with new (incorrect) object-location associations and subjects indicated by button press if presented position is “correct.” In 3-AFC tests subjects specified by button press (“1,” “2,” or “3”) the correct position of the building shown above the schematic street map. **(B)** Timeline Training. Each of the two study blocks (cross-over design) comprised 6 sessions (2–7; first session (not shown) included baseline testing) with 3 months in-between. Training (session 3–5) consisted of three consecutive days, each comprised five learning blocks and subsequent cued recall test (IR, 3-AFC). Memory was also tested follow-up on session 6 (1-day) and 7 (1-month) post-training. Overnight retention (offline effects) was assessed by applying memory tests (IR, 3-AFC) before next training (session 4 and 5). In addition, at the beginning of each training day subjects self-rated their affective state (“Befindlichkeitsskalierung anhand von Kategorien und Eigenschaftswörtern”; BSKE, Janke et al., 2002), and provided information about their sleep (number of slept hours, sleep quality) of previous night. In pre- and post-training sessions (2,6,7) we asked for positive and negative affective state (PANAS, Watson et al., 1988) and applied additional memory tests to assess transfer effects in trained (LOCATO-15; shorter and less complex version of training paradigm, Kulzow et al., 2014), and untrained (Rey–Osterrieth Complex Figure (ROCF) Test, Knight and Kaplan, 2003; Rey Auditory Verbal Learning Test (AVLT), Helmstaedter et al., 2001) memory tasks. **(C)** Stimulation protocol. Anode (7 × 5 cm^2^) was attached to T6 (according to EEG 10–20 System) and return electrode (cathode: 10 × 10 cm^2^) contralateral above the eyebrow (supraorbital). Connector of the anode was positioned at the posterior edge distant from the return electrode. Larger size of the cathode renders the stimulation density functionally ineffective. Anodal transcranial direct current stimulation (atDCS) of 1 mA was administered during beginning of OLM-training (Session 3–5) for 20 min (“atDCS”) or 30 s (“sham”) and current was ramped up and down within 10 s. Abbreviations: IR, item recognition; 3-AFC, 3 alternative forced choice task; PANAS, Positive and Negative Affect Schedule; LOCATO-15, short version of object-location-memory task; ROCF, Rey–Osterrieth Complex Figure Test; AVLT, German version of the Rey Auditory Verbal Learning Test, d-day; mo, month; FU, Follow-up.

Figure 1 provides an overview of procedure and tasks (for details see text below). To train visuospatial memory the OLM-task called LOCATO was used (Floel et al., 2012; Kulzow et al., 2014). In the present study, 30 pictures of real-life buildings (objects) were associated with different positions (locations) on a two-dimensional street map (“LOCATO-30”). Subjects had to learn the correct object-location pairings within five learning blocks on each of three training days. In detail, each learning block contained 120 trials (2 × 30 correct and 60 incorrect object-location associations) presented in randomized order resulting in a total of 1,800 trials across 3-day training. Thus, over the course of 3-day training correct object-location pairings were shown 30 times (10 per day) more frequently compared to “incorrect” positions (shown only once, respectively). Each trial comprised a picture of a schematized street map with one building presented for 3,000 ms and an inter-stimulus interval of 1,000 ms. Within this time frame subjects had to indicate by button press (“YES,” “NO”) on a response pad as accurate as possible whether the building was presented at the “correct” or “incorrect” location (see Figure 1A). Correct/incorrect responses were recorded during each learning trial in every learning block. No online feedback on performance was provided. Memory performance was tested shortly after the end the of the fifth learning block. Performance was assessed by cued recall using two different test formats, namely item recognition (IR) and 3-alternative forced choice (3-AFC) test (see also “Recall Format” of Figure 1A). To avoid contaminations due to task order, and to reduce overall testing time, 50% of associations were tested via IR and the remaining 50% via 3-AFC. For IR, 15 correct object-location associations were intermixed with 15 new (not shown before) incorrect pairings. Stimulus presentation was identical to learning blocks and subjects had to indicate by button press if the position was “correct” (“YES”) or “incorrect” (“NO”), timing was self-paced. In the subsequent 3-AFC test three possible locations for a particular building were shown on the street map marked with “1,” “2,” and “3.” The subject had to choose the building's “correct” location by pressing the corresponding number on the keyboard, timing was self-paced. Two parallel versions (A,B) of LOCATO-30 were used, each with a different set of buildings, and with the street map rotated for 180° for version B. Versions were assigned in counterbalanced manner to respective intervention. LOCATO was presented on a computer using Presentation software (Neurobehavioral Systems, Albany, CA, USA).

### Transfer tasks

Transfer on trained function was measured by a LOCATO-15 (short version of training task). LOCATO-15 comprised object-location-learning (OLL) of 15 associations within three learning blocks on a less complex street-map compared to LOCATO-30 training task. Memory was tested immediately after learning by a 3-AFC cued recall test. Three parallel versions were used for pre- and FU1 and FU2 tests. The parallel versions consisted of different sets of buildings presented on different street maps, and were applied in balanced order across subjects and time points. Transfer effects on untrained memory functions were assessed by using the two following learning and memory tasks: (i) Rey–Osterrieth Complex Figure Test (ROCF; originally designed by Rey, 1941; Knight and Kaplan, 2003), which require to copy a complex figure from original and delayed (approx. 20 min) from memory, and (ii) German version of the Rey Auditory Verbal Learning Test (AVLT; Helmstaedter et al., 2001), which consisted of remembering 15 learned words after five immediate recall trials and again after a 30 min delay. For ROCF-Test and AVLT available parallel versions were used across subjects and time points to minimize test-retest effects, respectively.

### Brain stimulation

During the beginning of OLM-training, either atDCS (20 min of anodal tDCS, 1 mA) or sham (30 s of anodal tDCS, 1 mA) was applied in a ramp-like fashion (fade in and fade out 10 s, respectively). Stimulation was delivered by a direct current stimulator (NeuroConn GmbH, Ilmenau, Germany) using two saline-soaked surface sponge electrodes. As different configurations can significantly affect the resulting electrical field (e.g., Saturnino et al., 2015) set up of electrodes (position and orientation; see also Figure 1C) was precisely pre-defined by use of standard operation procedures. The anode (7 × 5 cm^2^, current density = 0.028 mA/cm^2^) was placed over right temporoparietal cortex, centered on T6 (according to the international 10–20 electroencephalography system). Another return electrode (cathode: 10 × 10 cm^2^, current density = 0.01 mA/cm^2^) was positioned contralateral above the left eyebrow (supraorbital) and was centered to the left eye pupil. Electrodes were attached to the scalp using rubber bands. Given that right temporoparietal region is implicated in the acquisition of OLM (Postma et al., 2008) and anodal tDCS over this area has been shown to improve performance on a similar version of the task employed in our study (Floel et al., 2012; Prehn et al., 2017) this site was selected for anodal simulation. Note, that the larger size of the cathode renders the stimulation density functionally ineffective. Moreover, the current density of cathode was below the required minimum (0.017 mA/cm^2^) to modify cortical excitability by tDCS in humans (Nitsche and Paulus, 2000; Nitsche et al., 2007). Perception of stimulation was prompted after application of atDCS or sham. Subjects had to indicate first, if they experienced the stimulation (YES/NO) and second, to rate their level of discomfort due to stimulation on a scale from 0 (not at all) to 6 (very strongly).

### Procedure

Procedure was identical for study block 1 and 2. OLM performance was tested immediately after the end of each training day (see Figure 1B). In addition, OLM performance was obtained at the next day before the respective intervention had started (to capture offline effects) and at FU1 and FU2 sessions. Moreover, before atDCS or sham was applied, potential confounders such as emotional state and sleep characteristics were assessed on every training day. Specific (e.g., anger, anxiety) and unspecific (e.g., activation, excitation) affective states (10 in total) were rated by means of the German questionnaire BSKE (“Befindlichkeitsskalierung anhand von Kategorien und Eigenschaftswörtern”; BSKE, Janke et al., 2002) on scales ranging from 0 (not at all) to 6 (very strongly). Subjective perception of sleep quality and sleep duration of prior night were determined by two questions, that is “How did you sleep last night?” [rated from 0 (lousy) to 6 (excellent)] and “How many hours did you sleep last night?” Besides performance measurement of trained and untrained memory functions (LOCATO-15, ROCF, AVLT), positive and negative affective state was self-rated by means of Positive and Negative Affect Schedule (PANAS, Watson et al., 1988) at pre- and FU1 and FU2 sessions. In contrast to aforementioned measurements quality of Life (WHO Quality of life, Angermeyer et al., 2000) and habitual subjective sleep quality (Pittsburgh Sleep Quality, Buysse et al., 1989) were controlled and assessed only once within the 1st study block by standardized questionnaires.

### Data aggregation

Percent correct scores (PC) were calculated for every learning block (L1-L5, acquisition) and IR-test (memory) on the basis of hits and correct rejections in the respective trial. PC was defined as follows: PC = [number of hits + number of correct rejections] ^*^ 100/total number of presented buildings. Performance in the other used memory test (3-AFC) was measured by number of correct selected responses in %. Primary outcome “training success” was pre-specified before start of study (see clinicaltrials.gov: NCT02110056). Training success was operationalized by PC at fifth learning block (L5) on last training day (day3) and adjusted for baseline performance in the very first learning block [PC_L5 day3_ − PC_L1 day1_] to account for inter-individual differences. Secondary outcome comprised memory after 1-month post-training (FU 2). Therefore, cued recall performance (3-AFC, IR) at FU 2 was used and adjusted for learning performance after training day 1 (PC_L5_). For exploratory analyses, we computed indices for *online* (within-session), and *offline* (between-session) performance. Online scores were related to improvements (difference) within each training day [ON_day n_ = PC_L5_day n_ − PC_L1_day n_], and offline scores to overnight changes in performance. Offline scores included cued recall performance (3-AFC and IR, respectively) before start of next OLM-training in relation to learning performance of previous day and were determined in the following way: OFF_day n_ = Cued recall_day n+1 before OLM−training_ − PC_L5_day n_.

### Statistics

All Statistical analyses were conducted with IBM SPSS 24 (www.ibm.com/software/de/analytics/spss/). Perception of stimulation between training with and without atDCS was compared by non-parametric Wilcoxon test. Categorical variables (stimulus assignment) were analyzed by chi-square-tests (χ^2^). The effect of atDCS concomitant to training was analyzed using separate linear mixed models (random intercept models; Verbeke and Molenberghs, 2000). To test effects on training success and delayed memory, repeated measurements (“atDCS” or “sham”) were entered as level one unit nested in different individuals as level two units (64 data points in total). The stimulation effect was tested using the dichotomous variable INTERVENTION (atDCS, sham). Additional conducted analyses (exploratory) comprised on- and offline effects, and changes in emotional state and sleep characteristics across 3-day-training. For those analyses a further factor (factor “DAY”) was added to the models (192 data points in total for each model). To statistically control for potential confounders such as age, gender, education, baseline performance (pre-training performance in LOCATO-15), sequence of intervention, and MWT-score, analyses were repeated with these variables as covariates. Pre- and post-training mood (PANAS) and changes in trained and untrained transfer tasks (LOCATO-15, ROCF, AVLT) as a function of intervention were analyzed by separate linear mixed models with three factors “INTERVENTION” (atDCS, sham), “TIME” (Baseline, FU1, FU2), and “SEQUENCE” (study block 1, study block 2). Impact of intervention was reported by regression coefficients β, 95% confidence interval [95% CI], and d as a measure of effect size if not otherwise mentioned. An effect size is typically defined as the ratio of a difference between treatment and control group means to a standard deviation (SD). According to Hedges (2007) an appropriate SD in mixed model analysis can be obtained from the square root of summed covariance parameters (residual and intercept) in order to combine both, within- and between-subject variance. Estimate of d were then calculated by the ratio of estimated margin mean difference to SD. If appropriate, model-based *post-hoc* tests were computed to specify effects using *post-hoc* margin mean differences and 95% CI of these differences. Mean differences in % were reported and refer to atDCS—sham, if not otherwise mentioned. No adjustments were made to correct for multiple comparisons. The two-sided level of significance for all analyses was set at α = 0.05.

## Results

### Memory outcomes from training

#### Training success and delayed memory

**Figure 2:**
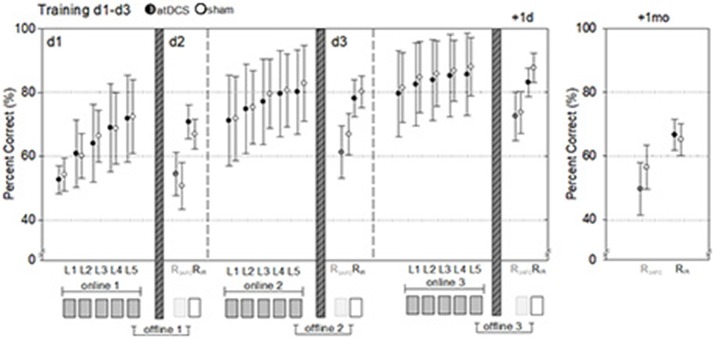
Performance during and after object-location-memory training. Response accuracy (% correct) during each learning (L) block and overnight cued recall performance (% correct) in 3-alternative forced choice (R_3AFC_) and item recognition (R_IR_) task assessed before next training on day 2 and 3 as well as cued recall at 1-day and 1-month follow-up is depicted. Dark filled circles (black, dark gray) represent performance of atDCS applied during training (“atDCS”), light filled circles represent performance of sham applied during training (“sham”). Behavioral online effects related to within-session performance and offline effects to between-session performance. Data are given as means and standard deviations. D, day; mo, month.

Learning and cued recall performance (3-AFC and IR) in LOCATO training task across days are presented in Figure 2. For “training success” linear mixed model analysis revealed no significant difference between atDCS relative to sham (−1.3 [−4.4, 1.9], *d* = 0.1). Also, no significant difference in delayed memory was found after previously administered atDCS compared to sham in neither of the used cued recall tests (3-AFC: −5.8 [−15.1, 3.4], *d* = 0.3; IR: 2.0 [−5.6, 9.5], *d* = 0.1). Linear mixed model analysis adjusted for the different covariates likewise did not show a significant beneficial atDCS effect on performance compared to sham (for details see Table 2).

**Table 2:** Results of separate linear mixed models analysis with factor “INTERVENTION” (atDCS vs. sham) for training success and delayed recall after 1-month (measured by 3-AFC and IR) without (MODEL 0) and with adjustment (MODEL 1).

**Outcome**	**Training success**			**Delayed recall (long-term memory)**					
				**3-AFC**			**IR**		
	***N***	**β**	**[95% CI]**	***N***	**β**	**[95% CI]**	***N***	**β**	**[95% CI]**
**MODEL 0**									
n (atDCS, sham)	32, 32			32, 30			31, 31		
Data points in total	64			62^*^			62^*^		
INTERVENTION		−1.3	[− 4.4,1.9]		−5.8	[−15.1, 3.4]		2.0	[−5.6, 9.5]
**MODEL 1 (ADJUSTED FOR AGE, EDUCATION, GENDER, BASELINE (LOC15), SEQUENCE, MWT)**									
n (atDCS, sham)	31,31			29,31			30,30		
Data points in total	62^*^			60^*^			60^*^		
INTERVENTION		−1.6	[−4.8, 1.7]		−5.3	[−14.5, 3.9]		1.9	[−5.3, 9.0]
Age		0.4	[−0.1, 0.9]		0.9	[−0.1, 1.8]		0.02	[−0.8, 0.8]
Education		−0.6	[−1.9, 0.8]		−0.02	[−2.2, 2.1]		−0.3	[−2.3, 1.8]
Gender		−6.8	[−14.9, 1.2]		−6.1	[−18.5, 6.2]		−1.8	[−13.2, 9.7]
Baseline LOC15		−0.3	[−0.7, 0.1]		0.02	[−0.6, 0.6]		0.3	[−0.2, 0.9]
Sequence		−1.9	[−5.2, 1.3]		7.9	[−1.4, 17.1]		9.1	[−2.0, 16.3]
MWT-score		−0.5	[−2.4, 1.4]		−0.1	[−3.1, 2.9]		0.7	[−2.1, 3.5]

*Three separate linear mixed models (MODEL 0: dependent variables: training success, 3-AFC and IR, respectively; independent variables: Intervention (atDCS, sham). MODEL 1 three separate linear mixed models with additional adjustment for age, education, gender, visuospatial baseline performance (LOC15), sequence of “atDCS” and “sham” and MWT (Multiple-Choice Vocabulary Intelligence Test)-score; n-number of individuals, β = regression coefficient (sham = 0); CI = confidence interval; 3-AFC, 3 alternative forced choice task; IR, item recognition; LOC15, LOCATO-15*.

* *Reduced data points due to missing data in training with atDCS or training with sham session*.

#### Behavioral measures of on- and offline effects

For between-session (offline) measurements significant less forgetting overnight after atDCS compared to sham were found after first night in IR test (4.2 [0.3, 8.0], *d* = 0.5), but not after night two (0.5 [−3.4, 4.4], *d* = 0.1) and three (−1.84 [−5.7,2.1], *d* = 0.2). This 1st night effect remained also after adjustment for covariates (4.1 [0.2, 8.0], *d* = 0.5). For 3 AFC-test this 1st night offline effect was less clear (4.1 [−2.0, 10.2], *d* = 0.3), but was also observable after adjustment for above mentioned confounders (5.4 [−0.6, 11.4], *d* = 0.4). Analysis of within-session (online) improvements revealed no significant effects of atDCS (see Table 3), but a substantial training effect was evident. Because subjects started on higher performance level each day, magnitude of online effects significantly decreased across training days (ON_day1_ = 18.8 [16.3, 21.0], *d* = 2.2; ON_day2_ = 9.9 [7.7, 12.1], *d* = 1.1; ON_day3_ = 6.3 [4.1, 8.5], d = 0.7).

**Table 3:** Mean Difference (Mean Diff) atDCS-sham and 95% CI of model based *post-hoc* comparisons between atDCS and sham for online (assessed as within-session difference performance score on each training day) and offline scores (assessed as overnight difference performance score between training sessions for 3-AFC and IR-scores) without (MODEL 0) and with adjustment (MODEL 1).

**Outcome**	**Online scores**			**Offline scores**					
				**3-AFC**			**IR**		
	***N***	**Mean Diff**	**[95% CI]**	***N***	**Mean Diff**	**[95% CI]**	***N***	**Mean Diff**	**[95% CI]**
**MODEL 0**									
n (atDCS, sham)	30, 32			32, 32			31, 32		
Data points in total	190^*^			192			191^*^		
Day 1		0.8	[−3.4, 5.1]		4.1	[−2.0, 10.2]		**4.2**	**[0.3, 8.0]**
Day 2		−2.2	[−2.2, 6.5]		−3.1	[−9.1, 3.0]		0.5	[−3.4, 4.4]
Day 3		−0.6	[−4.8, 3.7]		1.1	[−5.0, 7.2]		−1.84	[−5.7, 2.1]
**MODEL 1 (ADJUSTED FOR AGE, EDUCATION, GENDER, BASELINE (LOC15), SEQUENCE, MWT)**									
n (atDCS, sham)	30, 30			31, 31			31, 30		
Data points in total	184^*^			186^*^			185^*^		
Day 1		0.1	[−4.1, 4.4]		5.4^#^	[−0.6, 11.4]		**4.1**	**[0.2, 8.0]**
Day 2		−2.3	[−6.6, 2.0]		−1.6	[−7.6, 4.3]		0.7	[−3.2, 4.6]
Day 3		−0.4	[−4.7, 3.8]		1.9	[−4.1, 7.9]		−2.3	[−6.3, 1.6]

*Model-based post-hoc tests resulted from three separate linear mixed models (MODEL 0: dependent variables: online effects, offline effects for 3-AFC and IR, respectively; independent variables: Intervention (atDCS, sham), Day (d1,d2,d3) and Intervention x Day; MODEL 1 with additional adjustment for age, education, gender, visuospatial baseline performance (LOC15), sequence of “atDCS” and “sham" and MWT (Multiple-Choice Vocabulary Intelligence Test-score as covariates); n, number of individuals; CI, confidence interval; 3-AFC, 3 alternative forced choice task; IR, item recognition; LOC15, LOCATO-15*.

* *Reduced data points due to missing data in training with atDCS or training with sham session. Positive difference scores indicate better performance (online: better learning, offline: less overnight forgetting) of atDCS relative to sham. Significant differences (p < 0.05) are bold*.

# *p < 0.10*.

In sum, training success and delayed memory was not affected by atDCS, but 3-day visuospatial training significantly improved OLM independent of atDCS. A small benefit of atDCS relative to sham was restricted to the first offline score (after 1st night) as indicated by less overnight forgetting after receiving atDCS compared to sham.

### Control of sleep characteristics and affective state during training

Sleep characteristic did not significantly differ between atDCs and sham (for details see Table 4). Subjects slept on average 7 h and reported good quality of sleep of prior night (scored “4” on average on a scale from 0 (lousy) to 6 (excellent) across training days. Also, no significant differences were found with regard to positive (relaxation, good mood, activation, confidence) and negative (excitation, bad mood, anger, anxiety, depressed, deactivation) affective states between atDCS and sham rated immediately before beginning of each training (BSKE: F-statistics and associated *p*-values of fixed effects: all *p*'s > 0.08). Overall subjects felt rather positive and rated themselves very low on negative affective states (scores on average < 1; scale range: 0 “not at all” to 6 “very strongly”) across days.

**Table 4:** Mean difference (Mean Diff) and 95% CI of model based *post-hoc* comparisons (mixed model analysis between atDCS and sham (atDCS-sham); *n* = 31 individuals in training with atDCS sessions, *n* = 30 individuals in training with sham session; data points in total 187^*^) for reported sleep duration and sleep quality.

**Outcome**	**Sleep duration**		**Sleep quality**	
	**Mean Diff (atDCS-sham)**	**[95% CI]**	**Mean Diff (atDCS-sham)**	**[95% CI]**
Day 1	−0.1	[−0.5, 0.3]	0.1	[−0.2, 0.5]
Day 2	0.4	[−0.1, 0.8]	0.04	[−0.3, 0.4]
Day 3	0.2	[−0.2, 0.6]	0.3	[−0.7, 0.1]

*Model-based post-hoc tests resulted from two separate linear mixed models (MODEL: dependent variables: sleep duration, and sleep quality, respectively; independent variables: Intervention (atDCS, sham), Day (d1,d2,d3) and Intervention × Day. In both, atDCS and sham sessions, subjects slept 7 h on average across training days and reported good quality of sleep [scored “4” on average on a scale from 0 (lousy) to 6 (excellent)]*.

* *Reduced data points due to missing data*.

### Analysis of pre- and post-training tasks and mood

With regard to training gains on other tasks linear mixed model analysis with factors “INTERVENTION,” “TIME,” and “SEQUENCE” revealed no significant benefit of atDCS compared to sham neither for trained (LOCATO-15 task), nor for untrained (ROCF, AVLT) memory transfer tasks. However, a small inverse effect was observed for ROCF memory score (copy figure delayed from memory). Model based estimates indicated better performance 1-day after training for subjects receiving previously sham relative to atDCS (−2.9 [−5.1, −0.8], *d* = 0.4), but this difference did not persist after 1-month (−0.3 [−2.5, 1.8], *d* = 0.04). Although we used available parallel versions (three for LOCATO-15 and AVLT and two for ROCF) significant improvements in all outcomes of transfer tasks (except ROCF learning) were seen for performance in 2nd relative to 1st study block (significant “SEQUENCE” effects) probably due to practice and strategy learning during repeated testing.

With regard to mood no significant differences were found at pre- and FU1 and FU2 sessions between atDCS and sham. On average, subjects rated themselves higher on positive mood scale [mean (SD): 34.2(7.6)] than on negative mood scale [mean (SD): 11.3 (3.3)].

### Stimulation perception

Due to missing data, only data of 30 subjects were available for stimulation perception analysis. Analysis revealed that the majority of subjects could not discriminate atDCS from sham as 13 subjects believed to “never have received atDCS,” and 8 subjects reported that they received atDCS in both sessions. Only 9/30 subjects thought that they had received atDCS only once during the two study blocks, 7 of them assigned correctly and 2 incorrectly to the block comprising atDCS. However, cross-sectional analysis revealed that subjects neither in the first [$\chi_{\left(1\right)}^{2}$ = 0.4] nor in the second [$\chi_{\left(1\right)}^{2}$ = 2] study block could reliably differentiate stimulation conditions indicating that our sham procedure was successful in blinding subjects. Overall, subjects tolerated the procedure well. On average, subjects rated their “feeling of discomfort caused by stimulation” as very low on a scale ranging from 0 (not all) to 6 (extremely) under both, atDCS [mean (SD): 0.9 (1.2)] and sham [mean (SD): 0.7 (0.9)]. This difference was not significant (Z = −1.19).

## Discussion

The aim of this study was to investigate the effect of a combined intervention comprising 3 consecutive days of atDCS applied over right temporoparietal cortex and OLM-training in healthy older adults on training success, delayed memory (long-term effects after 1-month), and transfer tasks (generalization). First, we observed significant improvement in both training conditions, without additional gain induced by atDCS in training success or delayed memory performance. Second, exploratory analyses demonstrated a small benefit of atDCS for overnight forgetting rate, but this “offline” effect was confined to the first night. Third, results did not change after statistically controlling for a variety of proposed “modulators.” Forth, the combined intervention of atDCS and OLM-training did not promote performance on other trained and untrained memory tasks. In sum, in this cross-over study combining atDCS with an episodic memory training task (OLM), no improvement in the atDCS condition could be ascertained. However, a number of critical questions remain with regard to specifics of the experimental design, and also inter-individual differences in response to stimulation, which will be discussed. A more detailed understanding of potential modulators of the response to atDCS may help to boost episodic memory training with atDCS in older adults more successfully.

### Specifics of the experimental design

As suggested by Holland et al. (2011) a multi-day atDCS-training session protocol was applied, but in contrast to our recently reported results of beneficial effects of combined intervention on memory performance (Antonenko et al., 2017), we could not find a positive effect on training success. There are some decisive differences between both studies, which have to be considered when interpreting our findings. While Antonenko et al. (2017) aimed to study brain-behavior associations in a sample of young and older adults, we solely tested older adults, and age has been recognized as one of the factors affecting responsiveness to atDCS (Meinzer et al., 2013; Krause and Cohen Kadosh, 2014; Summers et al., 2016). The studies differed in additional parameters assumed to be relevant in atDCS/cognition modulating protocols (Shin et al., 2015) such as primary cognitive outcome, time point of assessment and selected statistical approach. Specifically, Antonenko et al. (2017) conducted resting state at baseline (pre-training) and with 1-day delay after OLM-training parallel to 3-AFC as recall test. In contrast, the present study determined “training success” by measuring performance (yes/no decision) during the last training block at third (last) training day. Both, the different test formats and time of testing (immediate vs. delayed) may involve different cognitive processes and physiological mechanisms of actions (Horvath et al., 2015) and could thus partly account for inconsistent results. Moreover, results from parallel group (Antonenko et al., 2017) may contradict results from within-group cross-over (present study) design, most notably because of temporal dynamics (carry-over effects). Most likely due to experience-based task-learning strategies, multi-day cognitive training resulted in strong practice effects during re-testing (cross-over design). Given that atDCS is a relatively weak form of modulation (Horvath et al., 2015), strong practice gains may have obscured subtle beneficial atDCS effects (see also Wang and Voss, 2015). Similarly, multi-day training would likely induce stronger practice effects during repeated testing than during single-session applications. This might explain different results between the present finding, and previous finding from our single-session approach (Floel et al., 2012). In addition, time course of atDCS after-effects are not fully understood and have not been systematically tested so far (Kuo et al., 2017). Together with possible carry-over effects, these after-effects might have masked beneficial effects of the stimulation condition on 1-month follow up testing. In sum, conceptual variability between different domains of cognition, known to be highly complex, have to be carefully taken into account when comparing studies. Further, more systematic work is required to understand temporal dynamics in multi-day cognitive training studies. For example, different experimental approaches should be employed in order to optimize methodological designs for atDCS-training approaches.

Alternatively, number of sessions may still have been too small to observe significant differences between conditions immediately after training or after 1-month. However, there is no consensus about minimum/maximum number of sessions and studies systematically comparing number of sessions are lacking. Accordingly, the number of applied training+atDCS sessions varies widely across studies (e.g., Berryhill, 2017), and no consistent picture emerged so far. For example, a 3-day training+atDCS approach has yielded positive (Talsma et al., 2016) but a 20-session protocol negative effects (Nilsson et al., 2017). Thus, it remains unclear if increase of number of sessions would have led to more pronounced differential findings for memory performance in the present study.

The lack of beneficial atDCS effects on memory performance could also be related to the used low intensity of 1 mA during stimulation. Note that non-linear intensity-dependent effects of stimulation has been demonstrated in the motor (e.g., Jamil et al., 2017), but also in the cognitive domain (Hoy et al., 2013). For both, the lower intensities showed equal, if not greater effects in motor-cortical excitability (greatest at 0.5 and 1.0 mA compared to 1.5 mA and 2 mA) and working memory (1 mA better than 2 mA), respectively. Thus, the use of higher current might not necessarily mean a greater impact on performance, but can even invert stimulation effects (Woods et al., 2016). Moreover, the use of a higher dose might be problematic, because of safety and tolerability reasons in the meaning of increasing the risk of side effects which could in turn negatively affect subject blinding. Given that we have previously found positive effects with the application of 1 mA on OLM (Floel et al., 2012; Antonenko et al., 2017; Prehn et al., 2017), alternative factors, e.g., timing in relation to task, interindividual differences, or a combination, might contribute to null finding.

An impact of repeated atDCS on overnight performance that emerge between training sessions—possibly via affecting consolidation processes—has been previously suggested (Reis et al., 2009, 2015). Similarly, we observed in our exploratory analysis at least a small atDCS related benefit on consolidation (“offline” effect), but after the first night only. Memory is a highly dynamic process (Schacter and Addis, 2007). After initial encoding memory traces are unstable (i.e., vulnerable to interference or modification), but become stabilized and more resistant to disruption over time, a process referred to as “consolidation” (McGaugh, 2000). Thus, the “offline” effect after the first night might indicate a relatively larger impact of atDCS on more “labile phases” of consolidation (Lally et al., 2013; Richmond et al., 2014). It is also consistent with the assumption that atDCS is most effective at near-threshold and fragile performance level (Berryhill et al., 2014). In contrast, Reis et al. (2009, 2015) reported increased offline skill gain over the course of five consecutive training days after receiving atDCS relative to sham. In fact, even consolidated memory can be returned into a transiently labile state again by reactivation (Lee, 2009; Dudai, 2012), e.g., by repeated retrieval, which then require another period of stabilization (reconsolidation; Sara, 2000; Nader and Hardt, 2009). However, given that the probability of being destabilized upon reactivation depend among others on strength of a memory (Dudai and Eisenberg, 2004; Bustos et al., 2009; Winters et al., 2009), susceptibility for modifications of a memory trace might change during training progress. Further, both training and atDCS implicate neuroplastic processes, which probably overlap in their underlying mechanisms, but may act on different time scales to boost task-related activity (Au et al., 2017). Besides, repeated sessions could affect intrinsic brain activity and might interact with training-induced plasticity (Moller et al., 2017). Therefore, non-optimal timing between those neuroplastic processes might have masked atDCS-related effects in the present study. Note also that optimal timing might differ for motor task and cognitive task (Summers et al., 2016). Even within the cognitive domain the optimal time gap between practice sessions seems to be moderated by the nature (here mental difficulty) of the task (Donovan and Radosevich, 1999). In sum, better synchronization of task- and stimulation-induced plasticity over training may help to improve atDCS impact in multi-day applications, a hypothesis to be tested in future studies. Moreover, *within*-session breaks of tDCS (spaced stimulation) may promote metaplasticity (Goldsworthy et al., 2015) and might also lead to longer-lasting effects. Hence, both spacing within- and between atDCS sessions seems to be critical in modulating neuroplasticity and should be carefully taken into account in designing multi-day atDCS training protocols.

### Individual differences

Profound variability amongst individuals in responsiveness to atDCS effects has been noted independent of protocol or electrode montage (e.g., Jantz et al., 2016). Accordingly, proposed moderators like gender, age, education, sequence of stimulation and baseline performance (e.g., Tseng et al., 2012; Meiron and Lavidor, 2013; Krause and Cohen Kadosh, 2014; Learmonth et al., 2015; Li et al., 2015; Santarnecchi et al., 2015; Hsu et al., 2016; Summers et al., 2016; Fertonani and Miniussi, 2017) were included in our statistical model. In addition, we considered variation in common genetic polymorphisms like APOE e4 carrier status (Elder and Taylor, 2014). However, no substantial impact on outcome of any of the selected “modulators” were found. Since intra-individual changes are known to increase with advanced age (Macdonald et al., 2006), we also monitored affective state (prior to each training) and sleep characteristics of the preceding night for each training+stimulation day, but found no differences between stimulation condition. However, most of the evidence related to inter-individual differences stems from single session studies, but both CT (Jaeggi et al., 2014; Katz et al., 2016) and CT in combination with atDCS can be potentially influenced by individual variability. Thus, it is important to further identify, control and/or counteract different sources of variability in multi-session studies, to allow for more reliable atDCS effects across individuals, see also (Shin et al., 2015; Davis, 2017) for further discussion.

### Effects on trained and untrained (transfer) material

The present study also explored the translational potential of combined intervention on trained (LOCATO-15; OLL) and untrained (verbal and visuo-constructive) memory functions. Previous studies using a combined atDCS+training approach had demonstrated beneficial impact on delayed parameters of the trained task and untrained functions, even in the absence of immediate effects (Jones et al., 2015; Wang and Voss, 2015; Stephens and Berryhill, 2016; Antonenko et al., 2017; Ruf et al., 2017). In contrast, we could not substantiate these positive findings, in line with other studies on transfer effects (see Elmasry et al., 2015; Nilsson et al., 2017). Several factors may account for our present results. First, training schedule may have been too short to modulate gains on other tasks, although OLM performance was high at the end of 3-day training (on average 87%). Further, performing successfully in trained task might motivate to perform well on other tasks following training (Hayes et al., 2015) overriding subtle atDCS induced modulations. Second, since we aimed to study augmentation of combined atDCS+training intervention we did not include a “atDCS only” control group which might be necessary to disentangle effects above and beyond training itself. Third, studies showing positive transfer effects used a parallel-group design. It is conceivable that complex carry-over effects due to cross-over design emerged that may have obscured statistical differences between stimulation and sham (for a similar discussion see also Wang and Voss, 2015). Because long-lasting transfer effects are highly relevant in the field of neurorehabilitation, careful design including appropriate control is needed to ascertain the mechanisms underlying successful transfer gains.

### Limitations

Several limitations should be acknowledged when interpreting these findings. First, we did not study oppose polarities. Consequently, using a cathodal tDCS control might provide more comprehensive information. However, the dichotomous approach of anodal/cathodal stimulation and associated improvement vs. impairment via increased/decreased neuronal excitability is mainly based on results of primary motor cortex stimulation studies (Berryhill et al., 2014), and might not apply to the cognitive function under study here. Second, only subjects were blinded to the stimulation condition. Although double-blind approach is considered as gold-standard, the risk of biasing subject's performance by investigator can be considered minor, because primary outcome was measured computerized. More importantly, the study was conducted in a randomized and sham controlled manner. Third, no modeling of current flow was employed (Chaieb et al., 2008). Thus, on the one hand stimulation site might not have been optimal for all subjects. However, the electrode-montage was not intended to induce focal stimulation. Instead, we aimed for stimulation of a cortical area connected to the fronto-hippocampal-parietal memory network sub serving the task under study (Nyberg, 2017). On the other hand, not only electrode site but also shape, size and connector position can interfere with the intensity and spatial distribution of the electric field generated around them. Direct measurements of electric fields are difficult to implement (Opitz et al., 2015). Nevertheless, compared to other tDCS studies the connectors were located relative far from each other (connector of the active electrode was positioned at the posterior edge and was relative distant from the return electrode). Hence, in line with the rough rule of thumb reported by Saturnino et al. (2015) the remote location of both connectors should result in strengthening the electric field in the brain region underneath the temporoparietal anode.

## Conclusion and outlook

tDCS, generally known to be a safe neuro-stimulation technique, was well-tolerated in healthy older subjects with stimulation and training-sessions over 3 consecutive days. As applied in its present form, the findings did not support the notion that the intervention combining atDCS and training improves memory formation in OLM. However, combined atDCS+training approach remains a fundamentally important goal in research on cognitive aging. Several factors may underlie the negative findings, as discussed in this report. Systematically addressing these factors in future studies may provide valuable information in order to advance in-depth knowledge in basic tDCS research to generate more robust results in individuals.

## Author contributions

NK contributed to conception, designed the study, performed analysis and interpretation, contributing to drafting and revising the paper; AVCdS contributed to acquisition, performed analysis and interpretation, drafting and revising the work; AF contributed to conception, designed the study, and critically revised the paper; UG contributed to analysis and interpretation of the data and revising the work; MC, J-MH, AG, SH, and SK contributed to acquisition and drafting of work; All authors provided final approval for the version to be published.

### Conflict of interest statement

The authors declare that the research was conducted in the absence of any commercial or financial relationships that could be construed as a potential conflict of interest.
